# Lipidomic profile and candidate biomarkers in septic patients

**DOI:** 10.1186/s12944-020-01246-2

**Published:** 2020-04-13

**Authors:** Giovana Colozza Mecatti, Márcia Cristina Fernandes Messias, Patrícia de Oliveira Carvalho

**Affiliations:** Laboratory of Multidisciplinary Research, São Francisco University, USF, São Francisco de Assis Avenue, 218, Bragança Paulista, SP 12916-900 Brazil

**Keywords:** Sepsis, Septic shock, Biomarker, Metabolomic, Lipidomic, Lysophosphatidylcoline

## Abstract

Sepsis is a severe disease with a high mortality rate. Identification and treatment in the initial hours of the disease improve outcomes. Some biomarkers like procalcitonin and C-reactive protein are used for diagnosis and to access sepsis prognosis and they can help in clinical decision-making, but none has sufficient specificity or sensitivity to be routinely employed in clinical practice. This review seeks to evaluate lipid metabolism alterations in patients with sepsis and the possibility of using the respective metabolites as biomarkers of the disease. A search of the main electronic biomedical databases was conducted for the 20-year period ending in February 2020, focused on primary research articles on biomarkers in sepsis. The keywords included sepsis, septic shock, biomarker, metabolomic, lipidomic and lysophosphatidylcoline.

. It concludes that altered lipid profiles, along with the progress of the disease should provide new insights, enabling a better understanding of the pathogenic mechanisms and making it possible to design new early diagnosis and therapeutic procedures for sepsis.

## Background

Sepsis is a major healthcare problem and affects millions of people around the world. Patients who develop the illness have high mortality rates (at least one in four) [1, 2]. Identification and treatment in the initial hours of the disease considerably improve outcomes [3]. Sepsis is a situation in which affected individuals develop an inflammatory response to an infection that harms their own organs and culminates in organ dysfunction [4]. Patients present signs of systemic inflammatory response syndrome and sometimes it is difficult for clinicians to define whether it is due to infection or other causes [5]. In that situation, the use of biomarkers could help with early diagnosis and improved risk stratification and clinical decision making [6–8].

Some biomarkers have been evaluated for use in sepsis diagnosis but none have sufficient specificity or sensitivity to be routinely employed in clinical practice. Procalcitonin (PCT) and C-reactive protein (CRP) have been the most widely used, but even they have limited ability to distinguish sepsis from other inflammatory conditions or to predict outcomes [9]. Altered lipid metabolism and its pro/anti-inflammatory lipid mediators play key roles in sepsis pathophysiology.

The use of multi-'omics’ (association of at least two ‘omic’ variables: genomic, lipidomic, proteomic or metabolomic) may lead to an understanding of the pathophysiology of the disease and to the development of appropriate therapeutics. For example, in the treatment cascade of an endotoxin, the performance of a drug could alter its neutralization, influencing clearance, inflammation, bacterial load and mortality [10].

The objective of this review is to evaluate the changes of lipid metabolism in patients with sepsis and the possibility of using the respective metabolites as biomarkers of this disease. A search of the main electronic biomedical databases (PubMed, MEDLINE, Scopus, and Web of Science) was conducted for the 20-year period ending in February 2020, focused on primary research articles on biomarkers in sepsis. The keywords searched for in the abstracts and titles included “sepsis”, “septic shock”, “biomarker,” “metabolomic”, “lipidomic”, and “lysophosphatidylcoline”. The search identified 16 references with the words lipidomic x sepsis x septic shock, 34 references with the words metabolomic x sepsis x septic shock, and 13 references with the words lysophosphatidylcoline x sepsis x septic shock.

## Review

### Sepsis as a healthcare problem

Sepsis is a major healthcare problem and affects millions of people around the world. The mortality rate among patients that develop the illness is high (at least one in four) [1, 2]. The actual incidence of the disease in the world is uncertain. However, Fleischmann et al. made a systematic bibliographic survey and based on a statistical extrapolation of the results obtained, they suggest estimates of 31.5 million cases ofinfection, 19.4 million cases of sepsis and 5.3 million deaths per year [11].

The incidence of sepsis is increasing annually. Analyses of the occurrence of sepsis over a 22-year period (1979 to 2000), using data of a nationally representative sample of hospitals in the United States, identified more than 10 million cases in 750 million hospitalizations [1]. A large observational study in a European setting revealed an estimated mean sepsis incidence of 212.7 cases per 100,000 inhabitants with an annual incidence increase of 7.3%. In Brazil, sepsis incidence was 36.3 per 1000 patient-days and sepsis mortality was 55.7% [12].

Sepsis is a syndrome involving factors of both pathogen and host such as age, comorbidities, environment and race. It is defined as a life-threatening organ dysfunction caused by a dysregulated host response to infection; a situation in which affected individuals develop an inflammatory response to an infection injuring their own organs and culminating in organ dysfunction. Septic shock is defined as a subset of sepsis in which underlying circulatory and cellular metabolism abnormalities are profound enough to substantially increase mortality. The patients present persistent hypotension requiring vasopressors to maintain mean arterial pressure ≥ 65 mmHg and hyperlactatemia in the absence of hypovolemia. In septic shock, mortality is more elevated (> 40%) and early identification and treatment in the initial hours of the disease improve outcomes [3].

Sepsis, similar to other systemic inflammatory response syndromes, is characterized by increased secretion of stress hormones (e.g. catecholamines and cortisol) and cytokines, complement system activation and mitochondrial dysfunction with decreased availability of ATP. Sepsis-related inflammation causes microcirculatory dysfunction, inadequate tissue oxygen supply and subcellular and cellular dysfunction [13]. Initially, in response to infection, the innate immunity is activated when microorganisms contact receptors localized in cell surfaces (toll-like receptors - TLR). Binding TLR stimulates intracellular signaling and, in turn, production of proinflammatory (TNF- α, IL-1) and anti-inflammatory molecules (IL-10) [14, 15]. There is an alteration to the pro-oxidant-antioxidant balance. Also, there is an increase in the concentration of inflammatory cytokines (TNF-α and IL-8) and a decrease in the plasma activity of superoxide dismutase (SOD) and catalase (CAT) [16]. TNF-α and IL-8 exert cardiac depression by reducing myocardial shortening [17], further jeopardizing the patient’s hemodynamics. Pro-inflammatory cytokines lead to larger adhesion molecules in neutrophils and endothelial cells. Activated neutrophils promote microorganism kill and injure endothelial cells too, increasing vascular permeability [18]. Cytokines foster coagulation, stimulating thrombin formation in the microvascular bed and contributing to organ failure. In addition, consumption of coagulation proteins promotes bleeding [15]. Organ failure may be explained by microvascular occlusion, disruption of oxygenation with tissue exudate and production of reactive oxygen types [19]. In addition, there is evidence that in sepsis, alterations occur in mitochondrial function, with a decrease in the supply of tissue oxygen thereby contributing to organic dysfunction and increased production of free radicals, impacting on cellular metabolism and inflammatory processes [20, 21]. The increase in the production of reactive oxygen species (ROS) leads to organic dysfunctions caused by cellular and endothelial lesion due to protein modification and lipid peroxidation [22].

### Effects of infection and inflammation on lipid and lipoprotein metabolism

Septic patients present alterations in lipid metabolism such as hypertriglyceridemia, a decrease in HDL and LDL-cholesterol and insulin resistance [23, 24]. Lipoprotein concentration can be reduced to 50% in patients with sepsis and those reductions seem to be related to the severity of the disease [25]. The primary decline is found in HDL and slow recovery occurs in HDL and LDL fractions. The decrease of HDL is not found in patients with trauma or other critical illnesses [26]. During sepsis, HDL is elevated in the HDL-acute phase attained in the presence of serum amyloid A, one of the three major acute phase proteins [27], and in depleted cholesterol and apolipoprotein A-1 [28] conditions. Inhibition of lipoprotein lipase, upregulated hepatic triglyceride production stimulated by hyperglycemia and hyperinsulinemia, the action of cytokines and the disruption of the synthesis-utilization balance are probably responsible for those alterations [29].

The changes in lipid metabolism during sepsis serve as a protective response against infection.

Lipopolysaccharide (LPS) is a constituent of Gram-negative bacteria that is involved in the inflammatory response to sepsis [30] and the presence of LPS in patients’ blood is a clear indicator of sepsis. However, detection of LPS in aqueous blood is complicated by the molecule’s amphiphilic biochemistry, which drives it to associate with host carrier lipoproteins [31] and other molecules such as LPS-binding protein (LBP), high-density lipoprotein (HDL), low-density lipoprotein (LDL), very low-density lipoprotein (VLDL) and bactericidal/permeability-increasing protein [32]. Lipoproteins are known to be involved in the response of immunity neutralizing LPS, reducing cellular adhesion and reducing inducible nitric oxide synthase expression [33]. The structural changes in HDL may have a protective function and have metabolic consequences [34]. Chylomicron and very low-density lipoprotein neutralize the biological effects of endotoxin, and HDL particles control infection and the systemic inflammatory response [29].

Inflammation is modulated by lipid mediators derived from long chain polyunsaturated fatty acids (PUFA) with 20 or 22 carbons (n-6 or n-3 families). Those lipid mediators (eicosanoids and docosanoids) lead to metabolic changes that alter the plasma FA profile [22]. Patients with sepsis present low concentrations of n-6 and n-3 PUFAs and a high n-6/n-3 ratio and that is associated with high mortality [35–38]. An increase in oleic acid (C18:1 *n*-9) accompanied by a decrease in the unsaturation index as well as in the levels of *n*-3 PUFA was observed in erythrocyte phospholipids of septic patients as compared to healthy controls [39]. Arachidonic acid metabolism is also markedly affected in patients with sepsis. A reduced LPS-induced release of AA and the COX-associated AA metabolites, 11-HETE, PGE2, and TXB2 was apparent in septic patients [40]. Also, decreased lysophosphatidylcholine (LPC) levels and increased ceramide (Cer) species rates in plasma are commonly associated with sepsis [37–41]. An investigation of sepsis from peritonitis using a swine model monitored changes in hemodynamic, blood chemistry, and inflammatory markers. Mass spectrometry-based targeted quantitative analyses of blood samples were performed and found marked decreased in PC and LPC species [42]. Those results were supported by our group in a clinical study which observed important alterations in lipid metabolism in patients with sepsis, specifically including LPCs and sphingomyelin (SMs). Both LPCs and SMs were downregulated, whereas the saturated and unsaturated PCs were upregulated in the plasma and erythrocytes of septic patients [39]. Previous studies have also demonstrated an increase in circulating phospholipase A2 type II (snp-PLA2) in patients with severe infection [43, 44]. Group IIA sPLA2 is an acute-phase protein that is expressed in various tissues and cells in response to pro-inflammatory cytokines and it serves to amplify the systemic inflammatory response [45]. Members of the sPLA2 family of enzymes generate bioactive lipid mediators that include lysophosphospholipids and arachidonic acid and which can be converted to eicosanoids. Eicosanoids modulate cell growth and differentiation, inflammation, immunity, platelet aggregation and many other functions. Eicosanoids produced from arachidonic acid by COX and LOX, respectively, are 2-series PG and 4-series LT that act as mediators of inflammatory processes [46].

### Biomarkers in sepsis

With the present systemic inflammatory response syndrome signals, it is sometimes difficult for clinicians to define whether it is due to infection or other causes [5]. In that situation, the use of biomarkers could help with early diagnosis, improving risk stratification and clinical decision-making [6–8].

Some biomarkers have been evaluated for use in sepsis. Most of them have been tested clinically, primarily as prognostic markers in sepsis. There are hundreds of biomarkers which could potentially be used for diagnosis and prognosis in septic patients [47]. They are classified as cytokine/chemokine biomarkers, cell marker biomarkers, receptor biomarkers, coagulation biomarkers, biomarkers related to vascular endothelial damage, biomarkers related to vasodilation, biomarkers of organ dysfunction and acute phase protein biomarkers [9]. Also, thirty-four biomarkers have been identified for use specifically in the diagnosis of sepsis but only five of them **(**CD11b**,** CD64**,** IL-12**,** IP-10 and PLA2-II) have reported sensitivity and specificity values greater than 90%.

A study with proteomic analysis, conducted with patients with sepsis and septic shock with a pulmonary focus, showed alterations in the proteins expressed in surviving and non-surviving sepsis patients alike. Of a total of 179, after excluding albumin and immunoglobulins, 48 were found to have been altered (16 specific proteins for survivors and 20 for non-survivors). Among the alterations in the concentrations of the proteins found were those associated to cytoskeletal organization, cell movement, energy metabolism, inflammation, coagulation and bleeding. The results also showed negative regulation of apolipoproteins like ApoA2, ApoA4, ApoC1, ApoC2, ApoC3, Apod and Pon1 [48].

So, due to their low specificity or sensitivity the use of these biomarkers is limited in routine clinical practice. Procalcitonin (PCT) and C-reactive protein (CRP) have been most widely used, but even they have limited ability to distinguish sepsis from other inflammatory conditions or to predict outcomes. Procalcitonin (PCT) is a propeptide of calcitonin produced in low concentrations by the thyroid, gastrointestinal tract and lungs in healthy individuals. In the presence of bacterial infections, pro-inflammatory mediators induce an upregulated production and, with treatment, levels decrease by 50% per day [49]. The use of PCT to guide antimicrobial therapy has low to moderate quality in minimizing endpoints like mortality, mechanical ventilation, clinical severity and reinfection [50].

Liu et al. conducted a meta-analysis with 86 articles and a total of 10,438 subjects included. They found descriptions of 60 biomarkers and the most common were procalcitonin, C-reactive protein, interleukin 6, soluble triggering receptor expressed on myeloid cells-1, presepsin, lipopolysaccharide binding protein and CD64. Plasma PCT, Strem-1 and presepsin had moderate diagnostic utility for indicating systemic response caused by infection rather than other causes [51]. C-reactive protein (CRP) and procalcitonin are the most commonly used biomarkers. However, CRP has low specificity [52]. Procalcitonin is more specific [53] than CPR, but it remains difficult for it to differentiate sepsis from other non-infection causes of inflammation [54].

A recent comprehensive review of the available experimental evidence has shown that different biomarkers have clearly been demonstrated as indicating varying injury mechanisms and can be used in early diagnosis for sepsis-induced acute kidney injury [55].

### Lipid biomarker

Lipids are regulators of cellular function and their metabolism is altered in patients with sepsis. Based on that, lipidomics can be used to understand the pathophysiological mechanisms involved in the diagnosis and the response to therapeutic measures [56]. Lipidomics is the analysis of lipid metabolism and is accessed by spectrophotometric techniques [57] and chromatography [58].

**Fig. 1:**
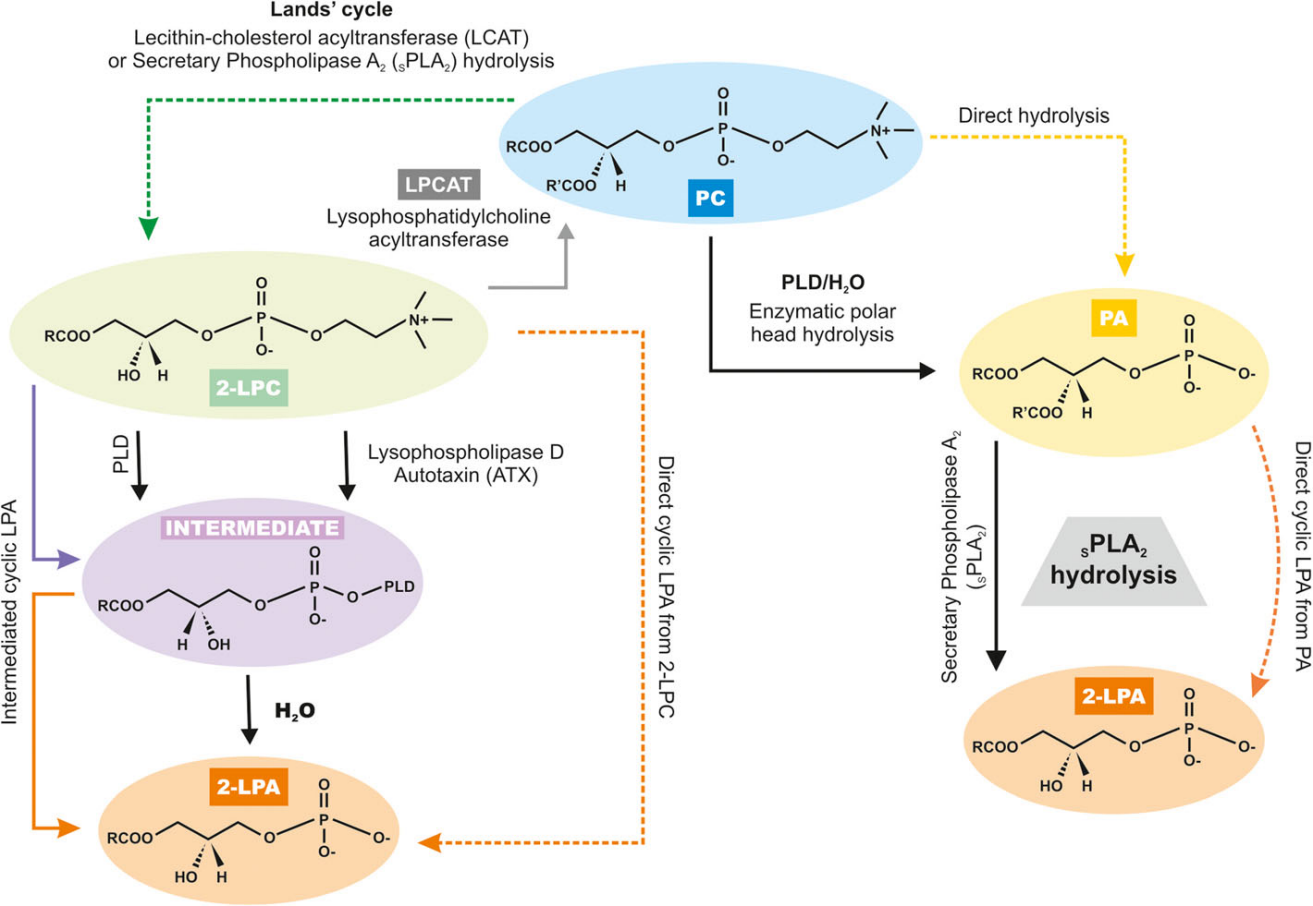
Metabolic pathways for LPC biosynthesis

LPC has been suggested to serve as a more useful prognostic marker for sepsis [37, 59, 60]. Park et al. performed a study comparing quantitative analyses of LPC 16:0 by using matrix-assisted laser desorption ionization time-of-flight (MALDI-TOF) mass spectrometry and found a sensitivity and a selectivity of medical diagnosis of sepsis estimated to be 97.9 and 95.5% on comparing analyses of sera from patients with severe sepsis and septic shock (*n* = 143), pneumonia patients (*n* = 12), and healthy individuals (*n* = 31) [61]. Lysophospholipids are membrane-derived phospholipids that can arise from homeostatic lipid metabolism or as a response to stimulus-induced cellular activation. Sources of plasma LPC include hydrolysis of PC by secretory phospholipase A2 (sPLA2) or lecithin:cholesterol acyltransferase (LCAT). LPC, in turn, is hydrolyzed to LPA in the plasma by autotaxin. LPA can also be synthesized from PA by sPLA2 [62]. In the phospholipid remodeling pathway, LPC is converted to PC via reacylation by acyl-CoA:lysophosphatidylcholine acyltransferase (LPCAT) in various tissues [63]. The schematic representation of the biosynthesis of LPC is represented in Fig. 1.

Total LPC concentration, as well as the concentration of the main LPC species, was markedly reduced in sepsis patients compared to controls and the difference in LPC-PC ratio was higher in survivors compared to non- survivors [37].

Cho et al., analyzed LPC concentration in blood samples on the first day after diagnosis of septic patients and compared them with a control group of healthy blood donors. The mean serum LPC concentration was significantly lower than in the healthy controls. No difference in serum LPC concentration was evident between survivors and non- survivors and no correlation was found with severity of the disease [59]. On the other hand a preliminary retrospective investigation of the analyses Ferrario et al. conducted of the plasma of 20 patients with septic shock found that decreases of unsaturated long-chain PC and LPC species were associated to the event at 28-days and 90-days, in combination with clinical variables such as cardiovascular SOFA score (28-day mortality model) or renal replacement therapy (90-day mortality model) [64].

In another study, authors evaluated serum LPC concentrations in patients in an emergency department with community-acquired pneumonia and correlated them to scores of clinical prediction indicators (pneumonia severity index (PSI) and CURB-65 score) and the concentration of procalcitonin. Samples of days 1 and 7 were analyzed. The mean LPC concentrations on days 1 and 7 were significantly lower in the non-survivors. Day 1 LPC concentrations were inversely correlated with the PSI and CURB- 65 scores. Day 1 LPC cut-off levels< 29.6 μmol/L were associated with the need for mechanical ventilation, vasopressors, intensive care unit admission, and hospital mortality [65]. Arshad et al. measured 105 phospholipids, 40 acylcarnitines, and 4 ceramides, as well as acid sphingomyelinase activity, in plasma from patients with community-acquired pneumonia, chronic obstructive pulmonary disease (COPD) exacerbation with infection and a control group, and found that Phospholipid concentrations were greatly decreased in community-acquired pneumonia and normalized in the course of clinical improvement. They also observed that changes in COPD were less pronounced, but also differed qualitatively [66].

The relation between serial LPC measurements with 28-day mortality was analyzed in a tertiary ICU in patients with sepsis and septic shock. Serum LPC, white blood cell, C-reactive protein and procalcitonin levels were measured at baseline (day 1 of admission) and day 7. The LPC concentration on day 7 was significantly lower in non-survivors compared to survivors and a decreased LPC concentration on day 7 and a sustained high concentration of procalcitonin on day 7 were useful for predicting the 28-day mortality. LPC concentrations increased over time in patients with appropriate antibiotics, but not in those with inappropriate antibiotics [67].

Other lipids have also been pointed out as possible sepsis markers. Ahn et al. evaluated alteration of the lipid profile of mice with sepsis induced by cecal bacterial peritonitis after ligature by cecal puncture. They observed that among 147 lipid species in the plasma, 13 subgroups (FA, LPA, LPC, LPE, PA, PC, PE, PI, MG, DG, TG, SM and Cer) had alterations in sepsis. The group also evaluated the response to administration of LPC and LPA with altered lipid profile in response [68].

Schmerler et al. demonstrate that acylcarnitines and glycerophosphatidylcholines may be helpful for differentiating infectious from non-infectious systemic inflammation due to their significantly higher concentration in sepsis patients [69]. Three lipids (PC(17:0/0:0, PE(P-19:1(12Z)/0:0), PE(22:2(13Z,16Z)/15:0),)) were selected to form a biomarker group to improve risk discrimination between the sepsis-induced lung injury patients and healthy cases [70].

Additional study design details in humans are listed in Table 1.

**Table 1:** Summary of Studies with evaluation of lipid biomarkers

Study	Methods	Participants	Interventions	Results
Drobnik et al., 2003 [37]	Prospective Experimental	102 patients with sepsis and 56 control	Analyses by mass spectrometry. The samples were collected as soon as sepsis criteria were met and mortality analyzed at 30 days.	Most Cer species were increased in sepsis patients, while all LPC species were markedly decreased. Species-specific as well as total Cer-SPM ratios were increased, whereas LPC-PC ratios were decreased in sepsis patients. The increased Cer-SPM ratios as well as the decreased LPC-PC ratios showed a strong predictive power for sepsis-related mortality. Total cholesterol, HDL-C, and LDL-C in sepsis patients were markedly reduced compared with a healthy population.
Cho et al., 2012 [59]	Prospective Not randomized	Patients meeting sepsis criteria (105) and control – healthy blood donors (21)	Blood samples collected on the first day. Samples were analyzed using the ANZWELL LPC Assay commercial kit (Alfresa Pharma Corporation, Osaka, Japan).	Mean of serum concentration of LPC was significantly lower in patients with sepsis than in healthy individuals. No differences were observed between survivors and non-survivors in septic patients.
Schmerler et al., 2012 [69]	Prospective Not randomized	161 patients (74 with SIRS, 69 with sepsis and 18 control – patients in ICU without SIRS)	Samples of blood samples were collected within the first 24 h of admission of patients with SIRS. For patients with sepsis, samples were collected at 24 h after the onset of organ dysfunction. Analyzes were performed by mass spectrometry.	Acylcarnitine (C10:1) and Phosphatidylcholine (PCaaC32:0) were significantly higher in patients with sepsis compared to patients with non-infectious SIRS.
Cho et al., 2014 [65]	Prospective Not randomized	A total of 56 patients with community-acquired pneumonia (CAP)	Blood samples were collected from patients with CAP on days 1 and 7 and analyzed for their plasma LPC concentrations. Blood samples were analyzed using an Anzwell LPC Assay Kit commercial (Alfresa Pharma, Osaka, Japan).	24 (42.9%) of patients required intubation and 15 (26.8%) died. The mean LPC concentrations on days 1 and 7 were significantly lower in the non-survivors. LPC levels < 29.6 μmol/L at day 1 were associated with outcomes such as the need for mechanical ventilation, vasopressors, ICU admission and hospital mortality.
Park et al., 2014 [67]	Prospective, observational	A total of 74 patients with confirmed diagnosis of infection with at least two criteria of SIRS, within 24 h of admission in ICU	Blood sample analyzed on day 1 and day 7. Blood samples were analyzed using an Anzwell LPC Assay Kit commercial (Alfresa Pharma, Osaka, Japan).	The LPC concentration on day 7 was significantly lower in non-survivors. A decreased LPC concentration on day 7 and sustained high concentration of procalcitonin on day 7 were related to 28-day mortality. LPC concentrations increased over time in patients with appropriate antibiotics.
Liang et al., 2016 [70]	Prospective	ICU patients with sepsis induced lung injury - SLI (80) and healthy volunteers (82)	Plasma samples were collected in the morning at ICU with 10 h of fasting and analyzed by chromatography/mass spectrometry.	Significant changes were found in 7 metabolites, with an increase in concentration in SLI patients in 5 of them and a decrease in 2. Lipid metabolites include PE (P-19: 1 (12Z) / 0: 0), PE (22: 2 13Z, 16Z) / 15: 0), PC (17: 0/0: 0), LPC (P-16: 0), PE (20: 3 (8Z, 11Z, 14Z) / 0: 16: 0/0: 0) and PC (17: 1 (10Z) / 0: 0). PE (P-19: 1 (12Z) / 0: 0) showed sensitivity of 98.1% and specificity of 97.3%. Three lipids (PE (P-19: 1 (12Z) / 0: 0), PE (22: 2 (13Z, 16Z) / 15: 0), PC (17: 0/0: 0)) were selected to form a group of biomarkers to improve risk discrimination among SIL patients and healthy cases.
Ferrario et al., 2016 [64]	Retrospective	Plasma of 20 patients with severe septic shock (SOFA score > 8) enrolled in a multicenter Study (Albumin Italian Outcome Sepsis Study)	Plasma samples were analyzed by spectrometry that included quantitative measurements of acylcarnitines, aminoacids, biogenicamines, glycerophospholipids, sphingolipids, and sugars.	Unsaturated long-chain phosphatidylcholines and LPC species were associated to the event at 28-days and 90-days in combination with clinical variables such as cardiovascular SOFA score (28-day mortality model) or renal replacement therapy (90-day mortality model).
Mecatti et al., 2018 [39]	Prospective Not randomized	Septic patients (n = 20) and healthy controls (*n* = 20)	Samples were collected in the first 36 h of admission to the ICU and analyzed by gas chromatography and mass spectrometry.	LPCs and SMs were downregulated, whereas the saturated and unsaturated phosphatidylcholines (PCs) were upregulated in the plasma and erythrocytes of septic patients.
Park et al., 2019 [61]	Prospective Controlled	Patients with severe sepsis and septic shock (*n* = 143), with pneumonia (*n* = 12), and healthy individuals (*n* = 31)	Quantitative analyses of LPC 16:0 were performed in samples of sera using a matrix-assisted laser desorption ionization time-of-flight (MALDI-TOF) mass spectrometry on a parylene-matrix chip.	Sensitivity of 97.9% and selectivity of 95.5% in sepsis diagnosis as compared to healthy individuals and patients with pneumonia.
Ferrario et al., 2019 [42]	Experimental Controlled	Swine model (*n* = 9)	Induced peritonitis was performed in a swine model, and changes in hemodynamic, blood chemistry, and inflammatory markers were monitored. Quantitative mass spectrometry-based targeted metabolomic analyses were performed.	Marked decrease in phosphatidylcholines and LPC species, altered alanine-glucose cycle and inter-organ amino acid metabolism.
Arshad et al., 2019 [66]	Prospective Controlled	Patients with community-acquired pneumonia (*n* = 29) and with chronic obstructive pulmonary disease exacerbation with infection (*n* = 13) and control group (*n* = 33)	105 phospholipids, 40 acylcarnitines, and 4 ceramides, as well as acid sphingomyelinase activity were analysed in plasma using a triple-quadrupole mass spectrometer.	Phospholipid concentrations were greatly decreased in community-acquired pneumonia and normalized in the course of clinical improvement. The changes in COPD were less pronounced, but also differed qualitatively.

*CRP* C-reactive protein, *ICU* intensive care unit, *LPC* lysophosphatidilcholine, *PCT* procalcitonin, *SIRS* systemic inflammatory response syndrome, *SLI* sepsis-induced lung injury

## Conclusion

In concluding this review, we can say that altered lipid profiles, along with the progress of diseases, should provide new insights that will enable a better understanding of the physiopathology of sepsis, contributing new possibilities for effective diagnoses and therapies. The current review deals with the lipid molecules that are up-regulated or down-regulated during the early stages of sepsis, as shown in the data presented in the present review and in earlier work of our research group [39]. Based on those aspects, we suggest that replenishing the protective molecules that are down-regulated in sepsis while withdrawing the elevated deleterious factors may lead to the discovery of new therapies for improving survival in septic patients; a goal that has been elusive for decades. In view of the complexity of the sepsis response, it is unlikely that a single ideal biomarker will ever be found. A combination of several sepsis biomarkers may be more effective, but that requires further evaluation.

## Data Availability

Not applicable.

## Abbreviations

11-HETE: 11-hydroxy-5,8,12,14-eicosatetraenoic acid
AA: Arachidonic acid
Apo: Apolipoprotein
CAT: Catalase
Cer: Ceramide
COX: Cyclooxygenase
CRP: C-reactive protein
DG: Diacylglyceride
DHA: Docosahexaenoic acid
FA: Fatty acid
HDL: High-density lipoprotein
IL: Interleukin
IP-10: Interferon gamma-induced protein 10
LDL: Low-density lipoprotein
LPA: Lysophosphate
LPC: Lysophosphatidylcholine
LPE: Lysophosphatidylethanolamine
PCT: Procalcitonin
PGE2: Prostaglandin E2
LOX: Lipoxygenase
LPS: Lipopolysaccharide
LT: Leukotriene
MG: Monoacylglyceride
PA: Phosphatidic acid
PC: Phosphatidylcholine
PE: Phosphatidiylethanolamine
PI: Phosphatidylinositol
PLA2-II: Phospholipase A2
PSI: Pneumonia severity index
PUFA: Polyunsaturated fatty acids
ROS: Reactive oxygen species
S1P: Sphingosine-1 phosphate
SM: Sphingomyelin
Snp-PLA2: Phospholipase A2 type II
Strem-1: Soluble triggering receptor expressed on myeloid cells-1
SOD: Superoxide dismutase
TG: Triacylglyceride
TLR: Toll-like receptors
TNF-alfa: Tumor necrosis factor alfa
TXB2: Thromboxane B2
VLDL: Very low-density lipoprotein
